# Advancing the Taxonomy of the Diatom *Pseudo-nitzschia* Through an Integrative Study Conducted in the Central and Southeastern Adriatic Sea

**DOI:** 10.3390/plants14020245

**Published:** 2025-01-16

**Authors:** Tina Bonačić, Jasna Arapov, Ivana Bušelić, Ivana Lepen Pleić, Blanka Milić Roje, Tina Tomašević, Mia Bužančić, Marija Mladinić, Silvia Casabianca, Antonella Penna, Sanda Skejić, Živana Ninčević Gladan

**Affiliations:** 1 Institute of Oceanography and Fisheries, Šetalište Ivana Meštrovića 63, 21000 Split, Croatia; arapov@izor.hr (J.A.); buselic@izor.hr (I.B.); lepen@izor.hr (I.L.P.); broje@izor.hr (B.M.R.); pavelin@izor.hr (T.T.); buzancic@izor.hr (M.B.); sanda@izor.hr (S.S.); nincevic@izor.hr (Ž.N.G.); 2 Doctoral Study of Biophysics, Faculty of Science, University of Split, Ruđera Boškovića 37, 21000 Split, Croatia; 3 Department of Biology, Faculty of Science, University of Zagreb, Ravnice 48, 10000 Zagreb, Croatia; marija.mladinic.m@gmail.com; 4 Department of Biomolecular Sciences, Campus E. Mattei, University of Urbino, Via Ca’ le Suore 2/4, 61029 Urbino, Italy; silvia.casabianca@uniurb.it (S.C.); antonella.penna@uniurb.it (A.P.); 5 CoNISMa National Inter-University Consortium for Marine Sciences, 00196 Rome, Italy

**Keywords:** diatoms, molecular analysis, morphology, ITS1-5.8S-ITS2, LSU, *rbc*L, FE-SEM/STEM, *Pseudo-nitzschia* spp., Bayesian inference, phylogeny

## Abstract

The marine diatom genus *Pseudo-nitzschia* comprises cosmopolitan phytoplankton species commonly present in the Adriatic Sea. Species within the genus *Pseudo-nitzschia* have been of significant concern because they produce domoic acid (DA), which can cause amnesic shellfish poisoning (ASP). In this study, we identified *Pseudo-nitzschia* species along the Central and Southeastern Adriatic Sea, where monthly sampling carried out from February 2022 to February 2024 allowed for comprehensive species documentation. *Pseudo-nitzschia* species cell cultures isolated from the study areas were morphologically and molecularly analysed. Morphological analyses were performed using a scanning electron microscope (FE-SEM/STEM), while molecular analyses were conducted, targeting the ITS1-5.8S-ITS2, LSU, and *rbc*L regions, to confirm species identity. This integrative approach led to the identification of eight species: *Pseudo-nitzschia allochrona*, *Pseudo-nitzschia calliantha*, *Pseudo-nitzschia delicatissima*, *Pseudo-nitzschia fraudulenta*, *Pseudo-nitzschia mannii*, *Pseudo-nitzschia multistriata*, *Pseudo-nitzschia pseudodelicatissima*, and *Pseudo-nitzschia subfraudulenta*. Our findings underscore the value of a combined approach for reliable species identification and contribute to the development of genetic sequence databases that support the advancement of next-generation methods such as metabarcoding. This research emphasises the importance of combined morphological and molecular methods for the differentiation of the cryptic and pseudo-cryptic *Pseudo-nitzschia* species.

## 1. Introduction

Diatoms of the genus *Pseudo-nitzschia* are well-known and extensively studied components of phytoplankton communities. Distinguishing these diatoms at the species level is extremely important from a public-health perspective, as some *Pseudo-nitzschia* species produce domoic acid (DA), a potent neurotoxin that can have harmful effects on humans and marine organisms [1,2]. Since 1987, *Pseudo-nitzschia* species have attracted considerable scientific attention following three reported deaths in Canada caused by the consumption of blue mussels containing high levels of DA, which resulted in amnesic shellfish poisoning (ASP) produced by the *Pseudo-nitzschia multiseries* [3]. Due to public health concerns, the number of described *Pseudo-nitzschia* species has increased rapidly. A 2012 study by Lelong et al. [1] reported that at least 37 species of the diatom genus *Pseudo-nitzschia* have been identified. Currently, there are 63 *Pseudo-nitzschia* species in total, out of which 28 species are known to produce DA [4,5,6,7,8,9].

However, the presence of cryptic and pseudo-cryptic species [10,11,12] within this genus limits the accuracy of traditionally used morphological methods and necessitates an integrative approach combining morphological and molecular data. Traditionally, *Pseudo-nitzschia* species can be categorised based on morphological characteristics into the *P. delicatissima* group, with a cell width less than 3 μm, and the *P. seriata* group, with a cell width greater than 3 μm. However, some *Pseudo-nitzschia* species, such as *P. multistriata* and *P. pungens*, fall into both categories because their cell widths overlap the defined morpho-categories.

Previous studies of *Pseudo-nitzschia* diversity along the Eastern Adriatic Sea generally focused on electron microscopy analyses [13,14,15,16,17,18]. Only a few studies included molecular sequencing related to the occurrence of *P. mannii* [19], *P. allochrona* (reported as *P.* cf. *arenysensis*) [20,21], and, from the latest report, *P. hasleana* and *P. galaxiae* [18]. Until now, integrative taxonomical approaches studying *Pseudo-nitzschia* diversity have been restricted to the Northern Adriatic Sea area [22,23,24].

The most used genetic markers for *Pseudo-nitzschia* identification are ITS1-5.8S-ITS2 and LSU [12], but new research is also employing the *rbc*L marker [23]. The use of various markers can aid in identifying cryptic and pseudo-cryptic species, such as *P. allochrona* or *P. delicatissima*, as well as *P. calliantha* or *P. pseudodelicatissima*, which belong to the *P. delicatissima* and *P. pseudodelicatissima* species complex and are common in the Adriatic Sea. The necessity of continuous research in the vicinity of mussel farms in the Adriatic Sea is also reflected in sporadic detection of low DA concentrations in this area [16,25]. Moreover, no integrated morphological and molecular data are available for the Central and Southern parts of the Eastern Adriatic Sea. Therefore, the main objective of this research was the morphological and molecular characterisation of *Pseudo-nitzschia* species in the coastal waters of the Central and Southeastern Adriatic Sea in the vicinity of mussel farms. The aim of this study was to develop a combined integrative database of morphological and molecular data on the *Pseudo-nitzschia* genus to enrich the publicly available ITS, LSU, and *rbc*L sequence databases and aid in distinguishing these cryptic and pseudo-cryptic species on a wider scale.

## 2. Results

During the study period, 321 *Pseudo-nitzschia* cell cultures were successfully established from the native phytoplankton community and molecularly sequenced at one gene marker. Out of all the established cell cultures, 184 were confirmed by sequencing at least two regions (ITS1-5.8S-ITS2 (hereafter referred to as ITS), LSU, or *rbc*L), and 113 were sequenced across all three regions: ITS, LSU, and *rbc*L (Supplementary Table S1). The *Pseudo-nitzschia* species included in this study were confirmed with a minimum of two barcodes (ITS, LSU, or *rbc*L), and 64 isolates were analysed with SEM in addition to molecular confirmation. Eight *Pseudo-nitzschia* species were established through molecular and morphological analyses of the isolated cell culture, namely, *P. allochrona*, *P. calliantha*, *P. delicatissima*, *P. fraudulenta*, *P. mannii*, *P. multistriata*, *P. pseudodelicatissima*, and *P. subfraudulenta*, with additional information regarding *rbc*L sequences for *P. hasleana* and *P. galaxiae* previously published in Arapov et al. [18]. The species that occurred in all the study locations were *P. calliantha* and *P. mannii*. The species *P. allochrona* and *P. delicatissima* were found at three study locations, as presented in Table 1. In contrast, the species *P. fraudulenta* and *P. pseudodelicatissima* were only isolated from Kaštela Bay, while *P. multistriata* was exclusively found in Šibenik Bay. Additionally, the presence of *P. subfraudulenta* was established in the Velebit Channel and Šibenik Bay.

The species *P. mannii* was the only species present throughout all the seasons, while *P. calliantha*, *P. delicatissima*, and *P. pseudodelicatissima* were successfully isolated throughout most of the year (Table 1). Species *P. allochrona* and *P. subfraudulenta* were successfully isolated during summer and autumn, while *P. fraudulenta* was isolated in autumn and winter. *P. multistriata* was only identified during autumn.

### 2.1. Morphological Characterisation

The morphological characteristics of the analysed *Pseudo-nitzschia* cell cultures are presented in Table 2.

**Figure 1:**
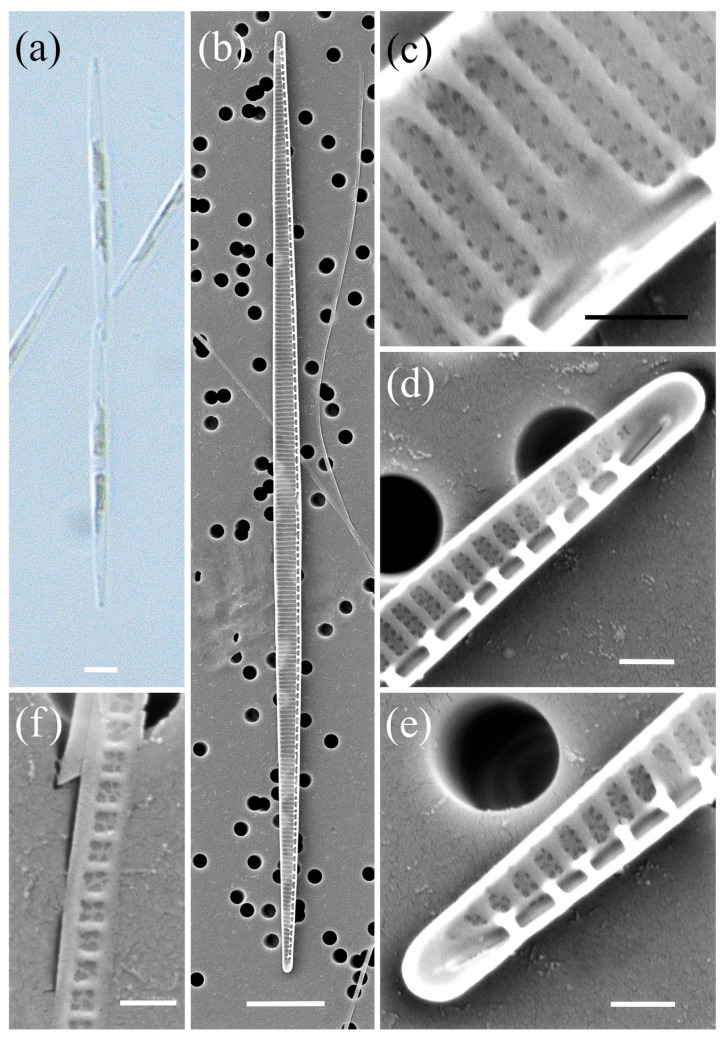
Micrographs of *Pseudo-nitzschia allochrona*: (**a**) colony in girdle view, LM; (**b**) whole valve, SEM; (**c**) central part of the valve face with central nodule, SEM; (**d**,**e**) valve ends, SEM; (**f**) girdle band with rows of perforations, SEM. Scale bars: (**a**,**b**) 5 µm; (**c**–**f**) 500 nm.

*Pseudo-nitzschia allochrona* Zingone, Percopo & Sarno (Figure 1, Table 2)

The cells were lanceolate with pointed ends in valve view. The valve length and valve width were between 51.06 and 63.01 µm and 1.44 and 2.15 μm, respectively. A central interspace with a central nodule, measuring 2–4 striae, was present. The density of fibulae ranged from 20 to 24, and that of the interstriae ranged from 36 to 39 in 10 µm. In general, each stria was perforated by two rows of poroids, and only at one valve was a single row of poroids observed close to the central nodule. The number of poroids in 1 µm varied from 7 to 13. The valvocopula contained 43 to 45 striae in 10 µm; these were one to two poroids high and two poroids wide.

**Figure 2:**
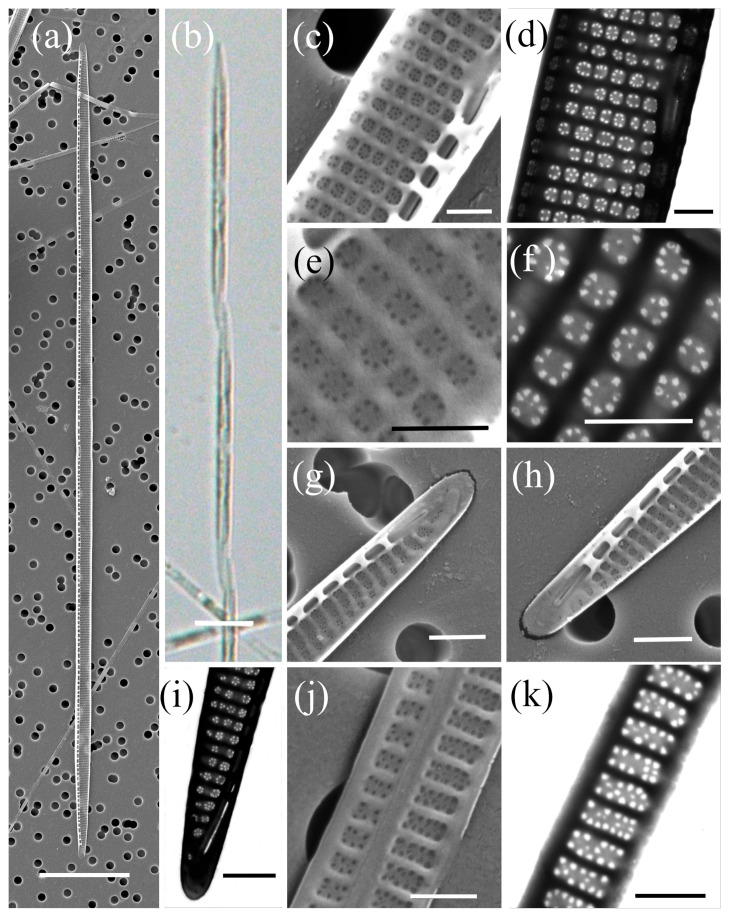
Micrographs of *Pseudo-nitzschia calliantha*: (**a**) whole valve, SEM; (**b**) colony in girdle view, LM; (**c**) central part of the valve face with central nodule, SEM; (**d**) central part of the valve face with central nodule, STEM; (**e**) poroid structure with sector detail, SEM; (**f**) poroid structure with sector detail, STEM; (**g**,**h**) valve ends, SEM; (**i**) valve end, STEM; (**j**) girdle band with rows of perforations, SEM; (**k**) girdle band with rows of perforations, STEM. Scale bars: (**a**,**b**) 10 µm; (**g**–**i**) 1 µm; (**c**–**f**,**j**,**k**) 500 nm.

*Pseudo-nitzschia calliantha* Lundholm, Moestrup & Hasle (Figure 2, Table 2)

The cells were linear in valve view, with a valve length ranging from 64.08 µm to 107.14 µm and a valve width ranging from 1.38 µm to 2.12 µm. A larger central interspace with a central nodule interrupting the raphe slit, was present and occupied three to five striae. The density of fibulae in 10 µm was between 17 and 22, and that of interstriae was between 34 and 38. The striae were uniseriate, with 4–6 round or square poroids in 1 µm. Each poroid was divided into 2–11 circularly arranged sectors, with a central sector found in 29% of the poroids (*n* = 3083). The majority of the poroids were divided into six (26%) and five sectors (24%), followed by seven (17%), four (16%), and eight (8%). The valvocopula contained 43–46 band striae in 10 µm, with structured striae measuring two to three poroids in width and three to six poroids (and rarely seven) in height.

**Figure 3:**
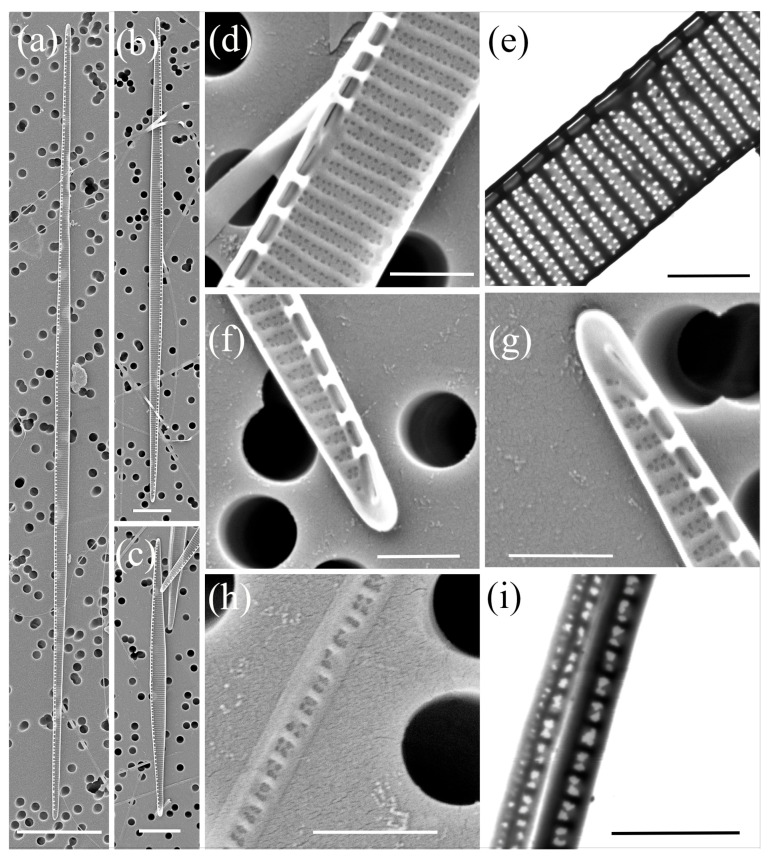
Micrographs of *Pseudo-nitzschia delicatissima*: (**a**–**c**) whole valve, SEM; (**d**) central part of the valve face with central nodule, SEM; (**e**) central part of the valve face with central nodule, STEM; (**f**,**g**) valve ends, SEM; (**h**) girdle band with rows of perforations, SEM; (**i**) girdle band with rows of perforations, STEM. Scale bars: (**a**) 10 µm; (**b**,**c**) 5 µm; (**d**–**i**) 1 µm.

*Pseudo-nitzschia delicatissima* (Cleve) Heiden (Figure 3, Table 2)

The cells were lanceolate with pointed ends in valve view. The valve length exhibited a large range from 31.05 to 94.21 µm, while the valve width ranged from 1.31 to 2.00 µm. A more pronounced lanceolate valve shape was observed in shorter cells, as they have similar widths regardless of the size of the apical axis. A central interspace with a central nodule was present and occupied 2 to 6 striae. The density of fibulae ranged from 20 to 26, and that of interstriae ranged from 37 to 42, in 10 µm. The striae were generally biseriate with 7–13 poroids in 1 µm, but uniseriate striae were also observed, albeit rarely. The valvovopula contained 48–51 band striae in 10 μm; these were one to two poroids high and wide.

**Figure 4:**
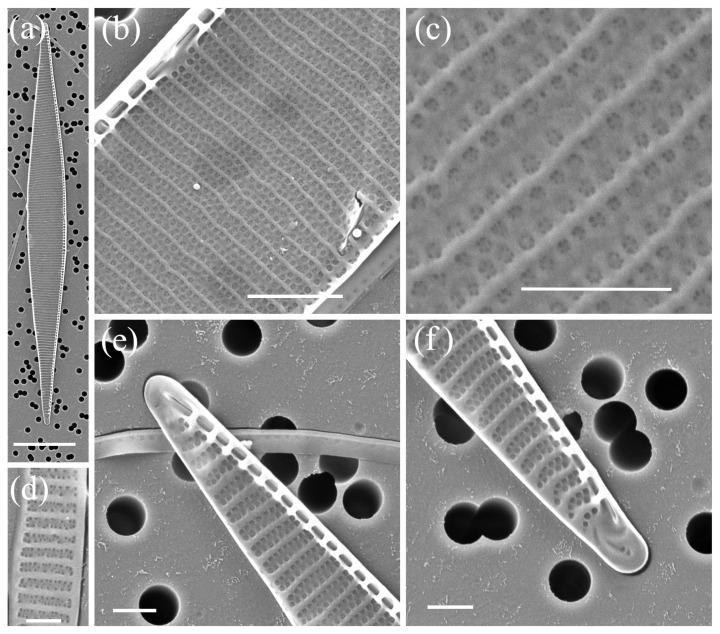
Micrographs of *Pseudo-nitzschia fraudulenta*: (**a**) whole valve, SEM; (**b**) central part of the valve face with central nodule, SEM; (**c**) poroid structure with sector detail, SEM; (**d**) girdle band with rows of perforations, SEM; (**e**,**f**) valve ends, SEM. Scale bars: (**a**) 10 µm; (**b**) 2 µm; (**c**,**e**,**f**) 1 µm; (**d**) 500 nm.

*Pseudo-nitzschia fraudulenta* (Cleve) Hasle (Figure 4, Table 2)

In valve view, the cells were lanceolate with pointed ends. The valve length was between 60.22 and 67.42 µm, and the valve width between 5.09 and 6.54 µm. A central interspace with a central nodule, measuring 3–5 striae, was present. The number of fibulae in 10 µm was almost the same or slightly less than the density of interstriae and ranged from 19 to 24; in comparison, the density of interstriae ranged from 23 to 25. The striae consisted mainly of two rows of poroids, but occasionally one or three rows of poroids were also observed, with five to seven poroids in 1 µm. The valves were slightly silicified, and the exact number of sectors was somewhat difficult to distinguish accurately. Most poroids were divided by four sectors (40%), followed by five (32%), six (14%), and four sectors (12%). The number of band striae in valvocopulae in 10 µm ranged from 38 to 41, and each striae was two to three poroids wide and up to 13 poroids high.

**Figure 5:**
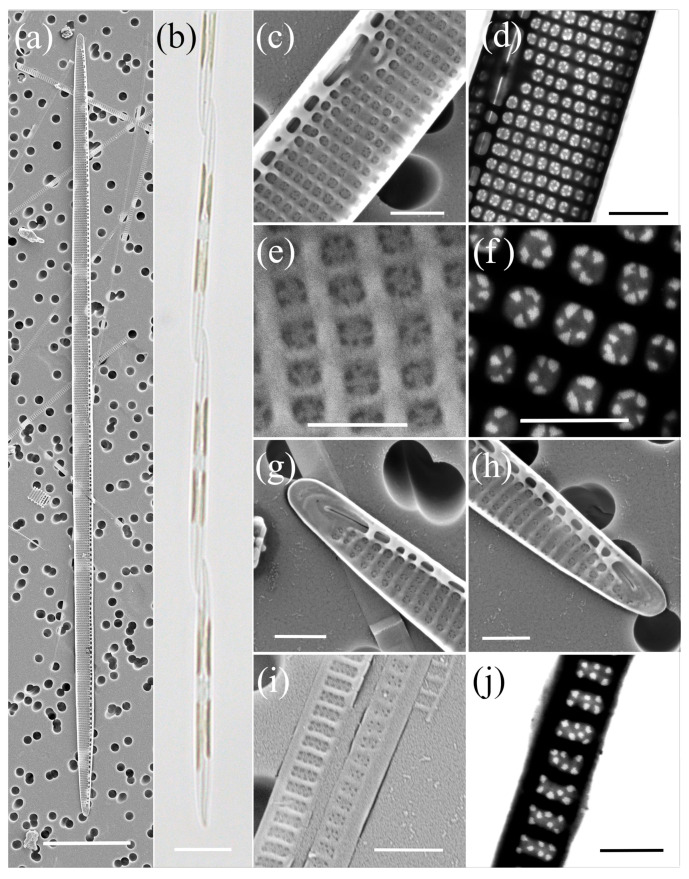
Micrographs of *Pseudo-nitzschia mannii*: (**a**) whole valve, SEM; (**b**) colony in girdle view, LM; (**c**) central part of the valve face with central nodule, SEM; (**d**) central part of the valve face with central nodule, STEM; (**e**) poroid structure with sector detail, SEM; (**f**) poroid structure with sector detail, STEM; (**g**,**h**) valve ends, SEM; (**i**) girdle band with rows of perforations, SEM; (**j**) girdle band with rows of perforations STEM. Scale bars: (**a**,**b**) 10 µm; (**c**,**d**) 1 µm; (**g**–**i**) 1 µm; (**e**,**f**,**j**) 500 nm.

*Pseudo-nitzschia mannii* Amato & Montresor (Figure 5, Table 2)

The valve length and valve width ranged from 69.08 to 123.56 μm and 1.71 to 2.48 μm, respectively. A central interspace with a central nodule was present and varied in size from two to six striae. The densities of the fibulae and interstriae were 16–23 and 33–37 in 10 μm, respectively. The striae were uniseriate with 4–6 poroids in 1 µm. The poroids consisted of one to eight sectors, predominantly four sectors (35%), followed by three (28%), five (17%), and two sectors (13%). The valvocopula contained 42–44 band striae in 10 μm; these were usually two poroids wide and up to five poroids high.

**Figure 6:**
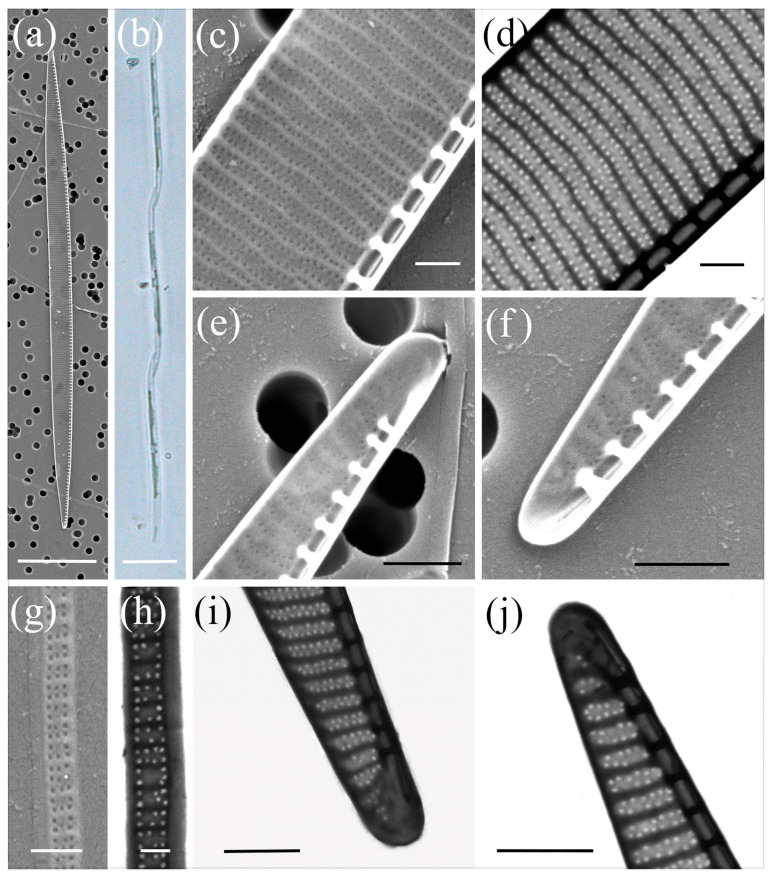
Micrographs of *Pseudo-nitzschia multistriata*: (**a**) whole valve, SEM; (**b**) colony in girdle view, LM; (**c**) central part of the valve face without central nodule, SEM; (**d**) central part of the valve face without central nodule, STEM; (**e**,**f**) valve ends, SEM; (**g**) girdle band with rows of perforations, SEM; (**h**) girdle band with rows of perforations, STEM; (**i**,**j**) valve ends, STEM. Scale bars: (**a**,**b**) 10 µm; (**e**,**f**,**i**,**j**) 1 µm; (**c**,**d**,**g**,**h**) 500 nm.

*Pseudo-nitzschia multistriata* H. Takano (Figure 6, Table 2)

The cells were lanceolate in valve view and sigmoidal in girdle view. The valve length and valve width were between 55.85 and 84.85 μm and 2.33 and 3.51 μm, respectively. A larger central interspace between the central fibulae including a nodule was not present. The striae were biseriate, and, rarely, one row of poroids was noticed. The density of unsegmented poroids ranged from 9 to 12 in 1 µm. The density of fibulae and interstriae in 10 µm ranged from 23 to 26 and 36 to 40, respectively. The valvocopula consisted of 47 to 51 band striae in 10 µm; these were two poroids wide and two to four poroids high.

**Figure 7:**
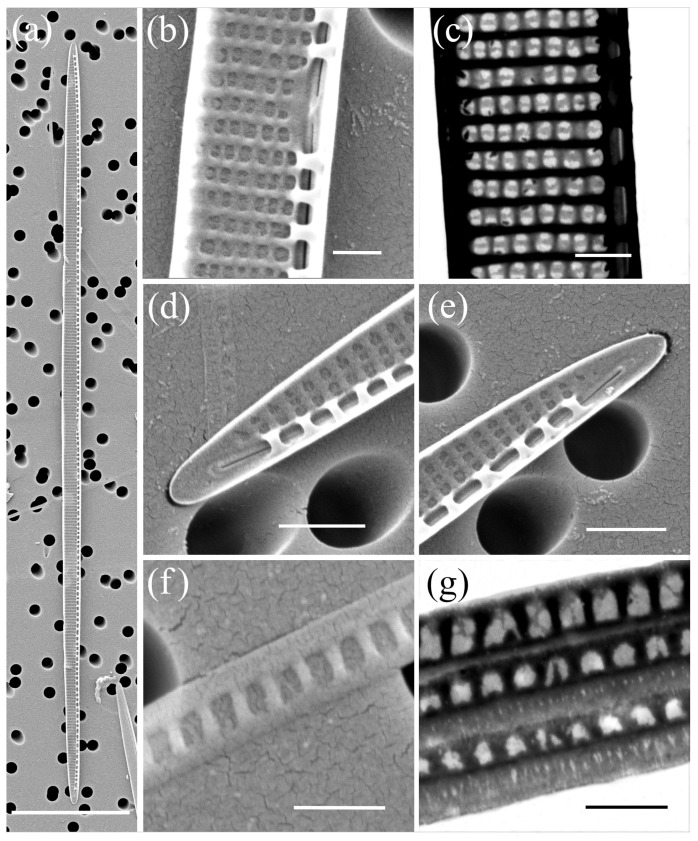
Micrographs of *Pseudo-nitzschia pseudodelicatissima*: (**a**) whole valve, SEM; (**b**) central part of the valve face with central nodule, SEM; (**c**) central part of the valve face with central nodule, STEM; (**d**,**e**) valve ends, SEM; (**f**) girdle band with rows of perforations, SEM; (**g**) girdle band with rows of perforations, STEM. Scale bars: (**a**) 10 µm; (**d**,**e**) 1 µm; (**b**,**c**,**f**,**g**) 500 nm.

*Pseudo-nitzschia pseudodelicatissima* (Hasle) Hasle (Figure 7, Table 2)

The cells were linear in valve view. The valve length and valve width ranged from 59.14 to 90.99 µm and 1.22 to 1.75 µm, respectively. A larger central interspace with a nodule was present and occupied three to seven striae. The density of fibulae and interstriae in 10 µm ranged from 19 to 26 and 38 to 41, respectively. The striae were uniseriate with a density of 5–7 oval to square poroids in 1 µm. The poroids were usually split into two large sectors (80%), but three (12%), one (4%), and four (3%) sectors were also observed. The number of band striae in valvocopulae ranged from 47 to 52.

**Figure 8:**
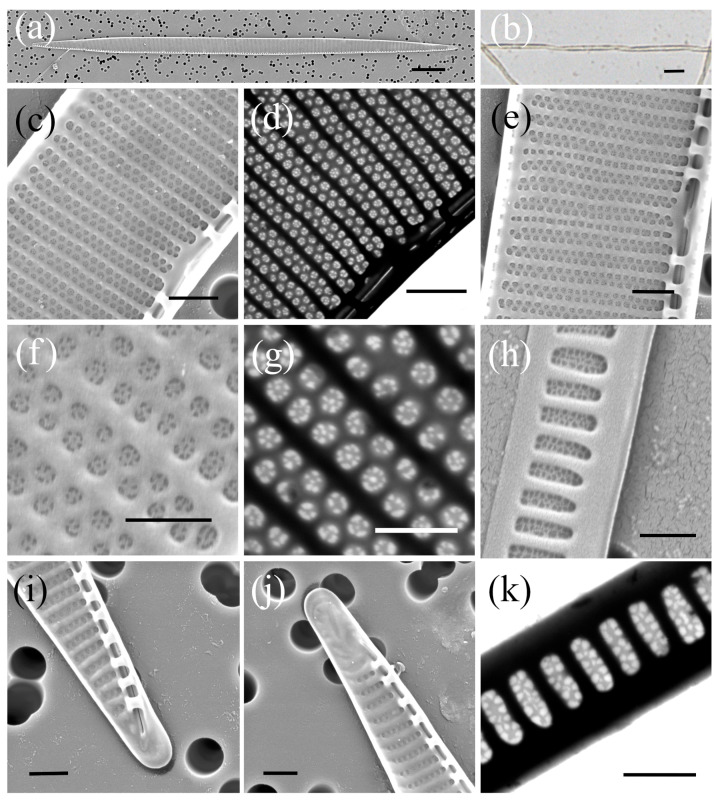
Micrographs of *Pseudo-nitzschia subfraudulenta*: (**a**) whole valve, SEM; (**b**) colony in girdle view, LM; (**c**) central part of the valve face with central nodule, SEM; (**d**) central part of the valve face with central nodule, STEM; (**e**) detail of the valve striae with one row of pores, SEM; (**f**) poroid structure with sector detail, SEM; (**g**) poroid structure with sector detail, STEM; (**h**) girdle band with rows of perforations, SEM; (**i**,**j**) valve ends, SEM; (**k**) girdle band with rows of perforations, STEM. Scale bars: (**a**,**b**) 10 µm; (**c**–**e**,**i**,**j**) 1 µm; (**f**–**h**,**k**) 500 nm.

*Pseudo-nitzschia subfraudulenta* (Hasle) Hasle (Figure 8, Table 2)

The cells were linear in the central part in valve view. The valve length ranged from 96.19 µm to 140.33 µm, and valve width ranged from 4.15 µm to 5.25 µm. A larger central interspace with a nodule was present, measuring five to six striae. The densities of fibulae and interstriae in 10 µm were 13–17 and 23–25, respectively. The striae were generally biseriate, although uniseriate striae were occasionally observed. There were 5–6 poroids in 1 µm. The poroids were divided into 2–10 sectors, with predominantly 4 (33%) and 5 sectors (26%) observed, followed by 6 (16%), 3 (14%), and 7 (7%) sectors. The valvocopula consisted of 39 to 41 band striae in 10 µm. In general, the band striae were two poroids wide (and rarely one, three, and four) and up to ten poroids high.

### 2.2. Phylogenetic Analyses Based on ITS, LSU, and rbcL

Trees were constructed using phylogenies based on ITS, LSU, and *rbc*L markers. The sequences from our study are noted in Supplementary Table S1 with corresponding species names, isolation locations, and GenBank accession numbers. The datasets yielded 127, 137, and 114 sequences for ITS, LSU, and *rbc*L, respectively. Alignments included 738 bp for the ITS alignment, 712 bp for the LSU alignment, and 1365 bp for the *rbc*L alignment. The overall average p-distances were as follows: ITS: 0.212, LSU: 0.029, and *rbc*L: 0.041; these results indicate that ITS was the most divergent marker of all. The genetically close species *P. mannii* and *P. calliantha* were very well resolved in all the observed phylogenetic trees, with strong nodal support > 0.94, while intraspecific variation within *P. calliantha* and *P. mannii* species was observed in the ITS and *rbc*L phylogenetic trees.

Variation was present within species isolated at the same location as well as between species isolated at different locations. *P. hasleana* is genetically and evolutionary close to *P. mannii* and *P. calliantha*, as evidenced in all three phylogenies, with an observed posterior probability of > 0.95. Nevertheless, there are clear differences between them. Slight intraspecific variation between native isolates of *P. hasleana* and imported sequences is noticeable in the ITS tree.

All the phylogenetic trees separated well the strains recorded in this study that are part of the pseudo-cryptic *Pseudo-nitzschia pseudodelicatissima* species complex. However, little data were available for *rbc*L phylogeny, and the ITS and LSU trees yielded the best results for *P. pseudodelicatissima, P. plurisecta* (present only in the ITS tree), *P. lundholmiae*, and *P. fukuyoi.* Overall, most intraspecific variations were observed in the *rbc*L phylogenies for *P. multistriata*, *P. fraudulenta*, *P. allochrona*, *P. delicatissima*, and *P. galaxiae*.

The species *P. allochrona* and *P. delicatissima* showed significant phylogenetic similarities and were well resolved in all barcodes, with strong posterior probability. All the phylogenetic trees in this study distinguished the cryptic species of *Pseudo-nitzschia delicatissima* complex well and with strong nodal support. Intraspecific variation among *P. allochrona* was detected in the *rbc*L tree. The observed variation was noted between isolates from the same study area and period (Šibenik Bay, September 2021). The mean p-distance for *P. allochrona* and *P. delicatissima* is 0.019. Low genetic distance is a confirmation of a morphologically similar but genetically distinct cryptic complex.

**Figure 9:**
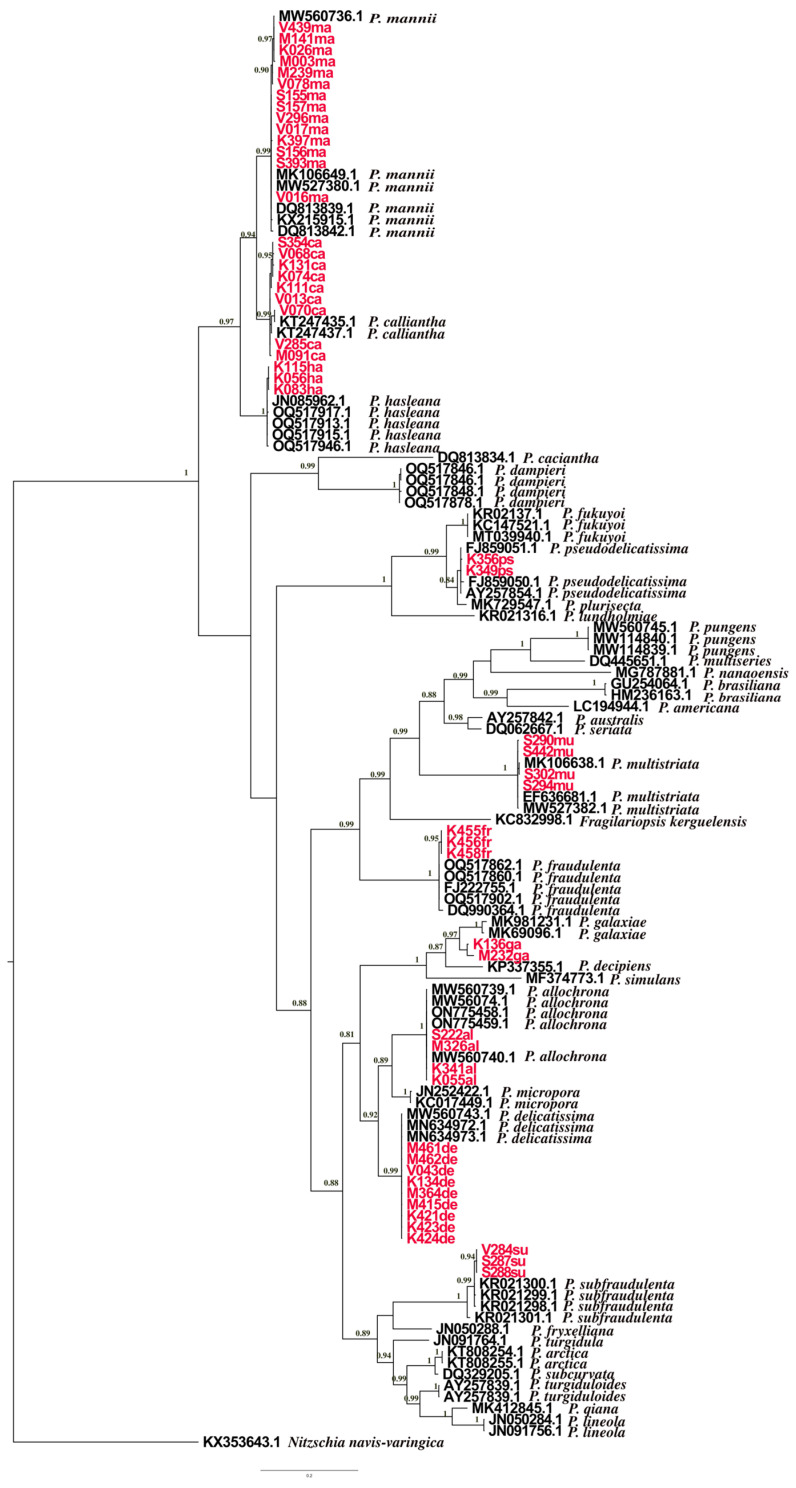
Phylogenetic tree reconstruction created using Bayesian tree based on ITS marker GTR+G+I; ngen = 5,000,000 Monte Carlo Markov Chain generations. Bayesian inference (BI) posterior probabilities (PP) > 0.90 are shown. Isolates sequenced in this study are presented in red. The scale bar represents 0.2 substitutions per site.

**Figure 10:**
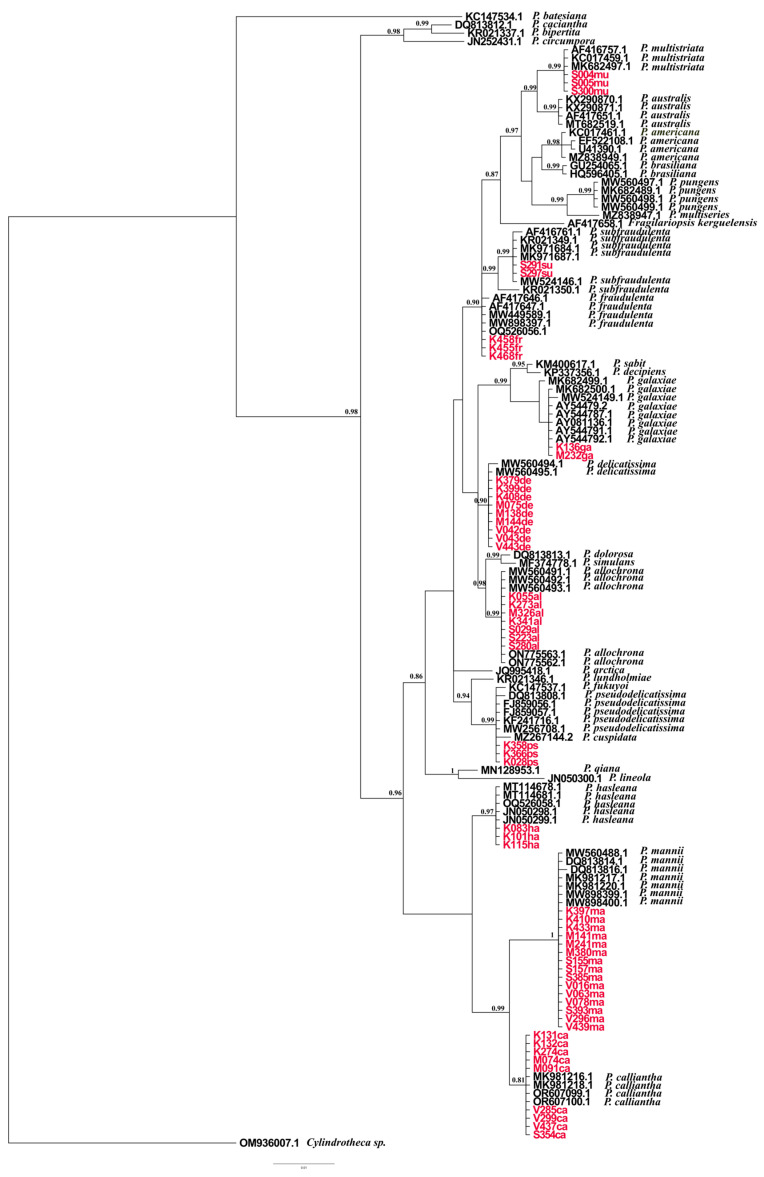
Phylogenetic tree reconstruction created using Bayesian tree based on LSU sequences GTR+G+I; ngen = 4,000,000 Monte Carlo Markov Chain generations. Bayesian inference (BI) posterior probabilities (PP) > 0.90 are shown. Isolates sequenced in this study are presented in red. The scale bar represents 0.01 substitutions per site.

**Figure 11:**
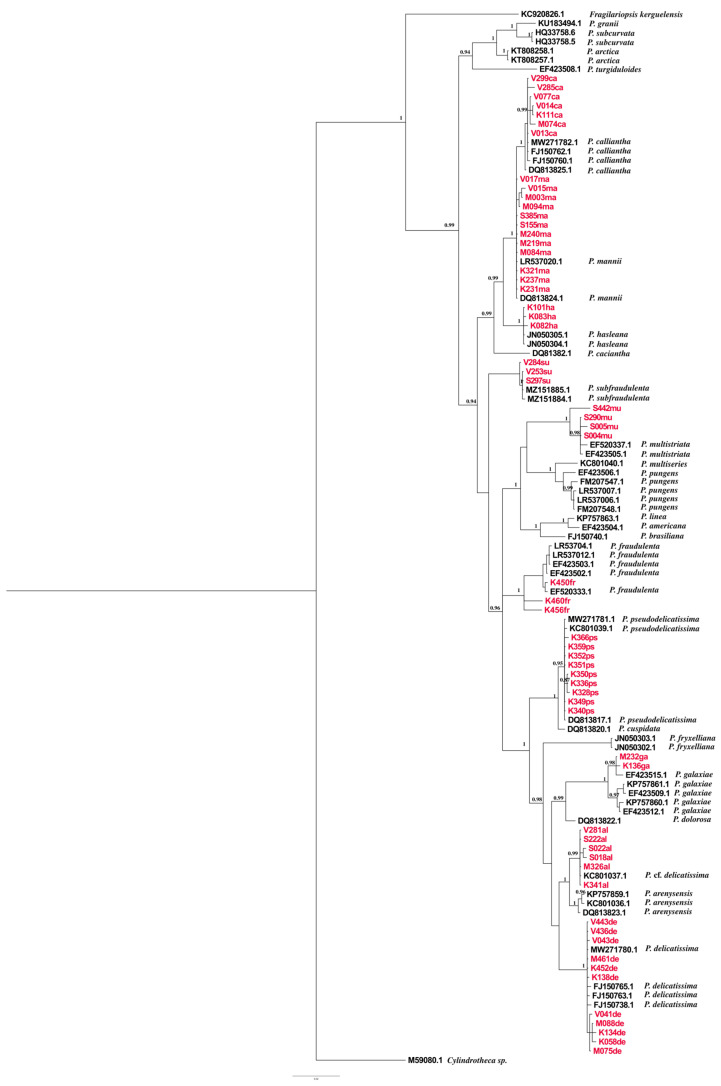
Phylogenetic tree reconstruction using Bayesian tree based on *rbc*L sequences GTR+G+I; ngen = 4,000,000 Monte Carlo Markov Chain generations. Bayesian inference (BI) posterior probabilities (PP) > 0.90 are shown. Isolates sequenced in this study are presented in red. The scale bar represents 0.02 substitutions per site.

Intraspecific variation between strains was observed in the ITS, LSU, and *rbc*L phylogenetic trees (Figure 9, Figure 10 and Figure 11). However, only the ITS and *rbc*L phylogenies showed evident variation between isolates from this study. The observed isolates were from two different locations and seasons: K136ga was isolated in Kaštela Bay in April 2022, while M232ga was isolated in Mali Ston Bay in September 2022. The species *P. galaxiae* is closest to the *Pseudo-nitzschia delicatissima* species complex in all the studied trees.

In this study, *P. fraudulenta* and *P. subfraudulenta* were identified as sister taxa only in the LSU phylogenies, and intraspecific variation was observed between the *P. subfraudulenta* isolates from this study and imported strains from GenBank. However, interspecific variation was observed in the ITS and *rbc*L phylogenies. Variation was present between native and imported species of *P. fraudulenta* and *P. subfraudulenta* in the ITS marker, while the *rbc*L marker distinguished variation among all the observed species.

**Figure 12:**
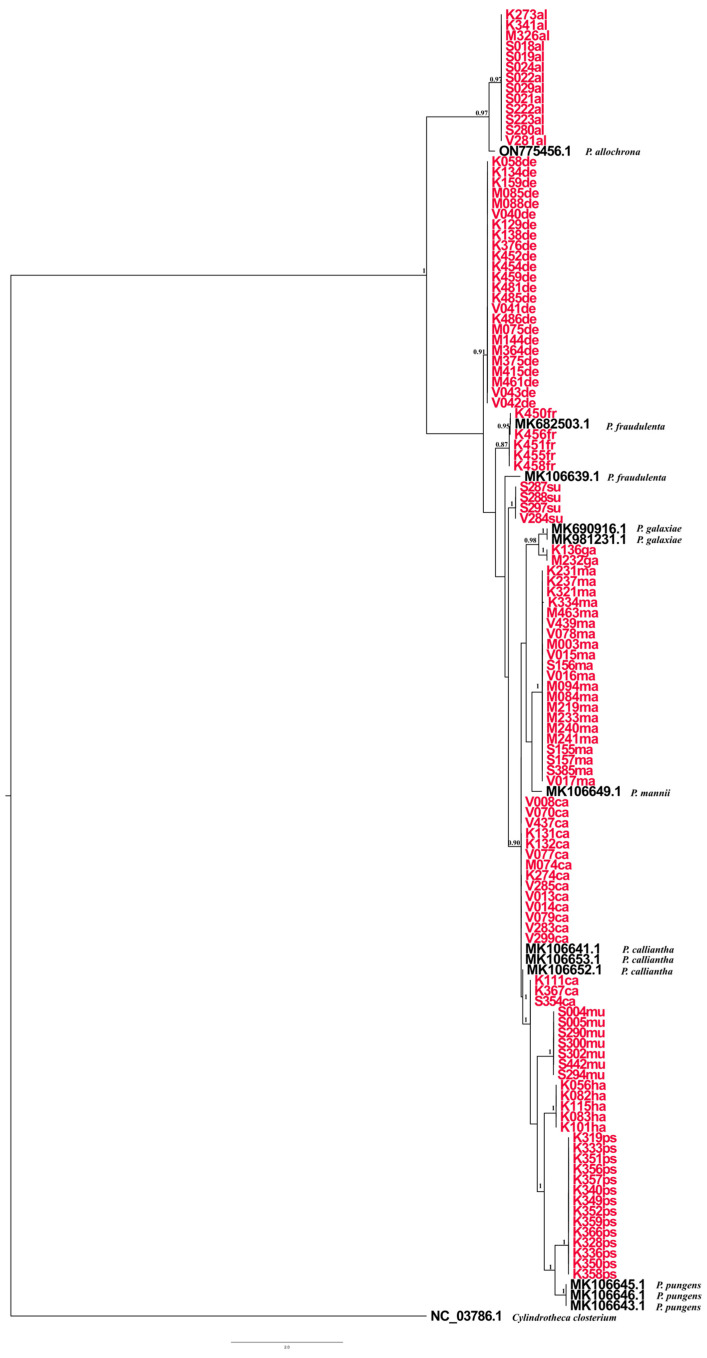
Combined phylogenetic tree reconstruction using Bayesian tree based on ITS, LSU, and *rbc*L marker GTR+G+I; ngen = 10,000,000 Monte Carlo Markov Chain generations. Bayesian inference (BI) posterior probabilities (PP) > 0.90 are shown. Isolates sequenced in this study are presented in red. The scale bar represents 1.0 substituions per site.

The species *P. multistriata* was found in all the phylogenetic trees to be part of the P. seriata group, which includes *P. australis*, *P. americana*, *P. brasiliana*, *P. nanaoensis*, *P. pungens*, *P. multiseries*, and *P. seriata*. A combined phylogenetic tree of *Pseudo-nitzschia* species was generated using BI analyses of ITS, LSU and *rbc*L gene regions, providing high-resolution insights (Figure 12). The combined phylogeny displays a well-resolved branching structure with high posterior probability values across major nodes. The generated structure reflects evolutionary divergence within the genus, with monophyletic groupings demonstrating clear phylogenetic separation among clades (*P. allochrona*, *P. delicatissima*, *P. fraudulenta*, *P. subfraudulenta*, *P. galaxiae*, *P. mannii*, *P. calliantha*, *P. multistriata*, *P. hasleana*, and *P. pseudodelicatissima*). The phylogenetic tree exhibits high-quality alignment across imported and native *Pseudo-nitzschia* sequences.

## 3. Discussion

This research presents the first comprehensive study of the genus *Pseudo-nitzschia* that combines molecular and morphological analyses conducted in the Central and Southeastern Adriatic Sea. By the end of the study period, 184 *Pseudo-nitzschia* cell cultures had successfully been isolated from the studied areas and further analysed morphologically and molecularly by using at least two genetic markers. Eight species were identified: *P. allochrona*, *P. calliantha*, *P. delicatissima*, *P. fraudulenta*, *P. mannii*, *P. multistriata*, *P. pseudodelicatissima*, and *P. subfraudulenta*. In this study, the species *P. subfraudulenta*, *P. fraudulenta*, and *P. multistriata* were confirmed molecularly using three barcodes after having been previously confirmed based on morphological/SEM analyses from the studied areas [20]. The species *P. hasleana* and *P. galaxiae* were additionally confirmed via sequencing the *rbc*L gene marker after they had previously been recorded on the ITS and LSU gene markers [16,17]. The species of the *Pseudo-nitzschia* genus found in this study have been previously reported to be in other areas of the Adriatic Sea [22,23,24,26,27].

Analysis of cell cultures using morphological and molecular methods provides an incomplete insight into the diversity of natural populations, but it does provide valuable information on *Pseudo-nitzschia* species distribution. In our study, the species *P. calliantha* and *P. mannii* were found across all the studied locations, and *P. mannii* was isolated in all seasons. In addition to *P. mannii*, the species *P. calliantha*, *P. delicatissima*, and *P. pseudodelictaissima* were isolated throughout most of the year, indicating the eurivalent capacity of these species. In accordance with our findings, similar studies have shown that *P. calliantha* can be isolated throughout the entire year in the Northern Adriatic [23], while *P. mannii* can be isolated in all seasons except summer [24]. The species that were found at only one site were *P. multistriata* and *P. fraudulenta*. We observed that *P. fraudulenta* was identified exclusively in Kaštela Bay, and its presence was restricted to winter months. The presence of *P. fraudulenta* has been previously reported, based on morphology, in Kaštela Bay during the winter season [16]. In this study, the presence of *P. fraudulenta* was verified through molecular sequencing. In the Northern Adriatic, the presence of *P. fraudulenta* was also confirmed during the winter months [22,23,24]. The species *P. multistriata* was found only in Šibenik Bay. Although it has previously been reported to occur in this area in all seasons [17], in the current study, it was isolated only during autumn and winter, similar to what was observed regarding the Northern Adriatic [23].

**Figure 13:**
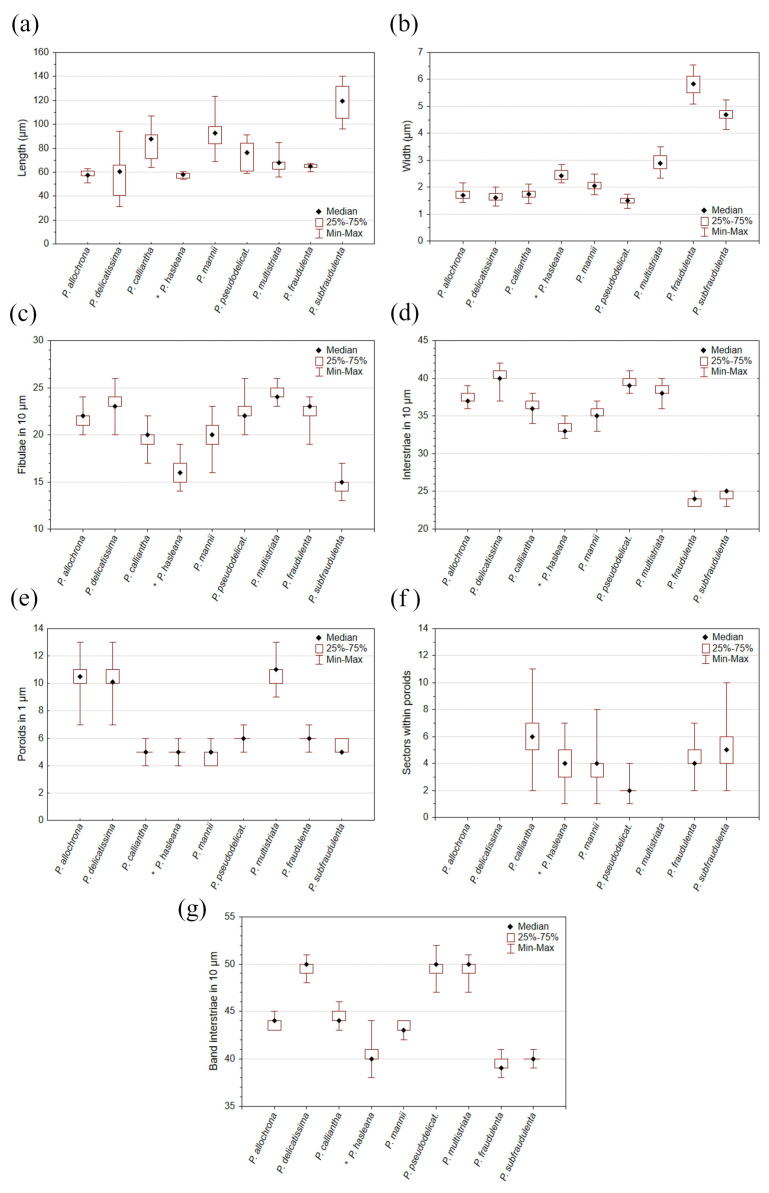
Morphometric data regarding observed *Pseudo-nitzschia* species: (**a**) length; (**b**) width; (**c**) number of fibulae in 10 µm; (**d**) number of interstriae in 10 µm; (**e**) number of poroids in 1 µm; (**f**) number of sectors within poroids; and (**g**) number of band interstriae in 10 µm.

Based on molecular data, two pseudo-cryptic species from the *Pseudo-nitzschia delicatissima* complex were distinguished, namely, *P. delicatissima* and *P. allochrona*, which were well recognised in all the analyzed phylogenetic trees. The only morphological difference between these species was observed in the number of band striae, with 43–45 band striae in 10 µm for *P. allochrona* and 48–51 for *P. delicatissima* (Figure 13). This result aligns with the previous findings regarding *P. allochrona*, which has 43–46 band striae, being identified as *P.* cf. *arenysensis* [20]. Similarly, Percopo et al. [21] reported 46–50 band striae, while Giulietti et al. [24] identified *P.* cf. *arenysensis* with a broader range of band striae (42–52). A lower number of band striae for *P. delicatissima* was reported by Lundholm et al. [28], ranging from 43 to 48. In our study, a different seasonal distribution between these cryptic species was noted, as *P. delicatissima* was isolated in all seasons except summer, contrary to the case for *P. allochrona*, which was isolated only in summer and autumn. This is in line with previous research, as *P. allochrona* has been found in the Mediterranean Sea during summer and autumn [20,21,24]. Pseudo-cryptic species from the *P. pseudodelicatissima* complex were differentiated based on molecular data, namely, *P. calliantha*, *P. mannii*, *P. hasleana*, and *P. pseudodelicatisisma*. In accordance with Smodlaka Tanković et al.’s research [29], the native strains of *P. pseudodelicatissima* from this study showed no variation among nucleotides for the *rbc*L barcode compared with Northern Adriatic strains (sequence MW271781.1, GenBank accession number). Although Lim et al. [30] stated that the separation of *P. cuspidata* and *P. pseudodelicatissima* remains unresolved, our *rbc*L phylogenetic tree showed visible intraspecific variation among them with a pairwise distance of 0.0051. This small distance of 0.0051 suggests that, on average, there is a 0.51% difference in the genetic material of the two species compared. A pairwise distance this small indicates that these two species are quite closely related and that they may have diverged relatively recently.

Morphologically, the species in the *P. pseudodelicatisisma* complex differed the most in terms of the number of sectors within the poroids and the number of band striae. The number of sectors was the highest for *P. calliantha*, ranging from 2 to 11, followed by *P. mannii*, with 1 to 8 sectors, and *P. pseudodelicatissima*, with the lowest number of sectors, ranging from 1 to 4 (Figure 13). *P. calliantha* and *P. mannii* overlapped in terms of all other morphological features, while *P. pseudodelicatisima* differed in regard to its higher number of band interstriae. In comparison with the mentioned species, *P. hasleana*, (previously described by Arapov et al. [18]) showed a slightly lower number of fibulae and interstriae, and a number of sectors were in between *P. calliantha* and *P. mannii*. In general, the species *P. pseudodelicatisisma*, *P. calliantha*, and *P. mannii* correspond to their original morphological descriptions [26,31].

Phylogenetic trees inferred from BI analyses of ITS, LSU, and *rbc*L revealed that the strains from this study clustered in 10 supported clades, with a posterior probability >0.90 (i.e., *P. allochrona*, *P. calliantha*, *P. delicatissima*, *P. fraudulenta*, *P. galaxiae*, *P. hasleana*, *P. mannii*, *P. multistriata*, *P. pseudodelicatissima*, and *P. subfraudulenta*). The overall topology of the phylogenetic trees was comparable, regardless of the marker used. ITS was the most divergent and variable marker, similar to what Turk Dermastia et al. [23] found for ITS2. As reported by Amato et al. [31], ITS is technically problematic because it occurs in multiple repeats in the genome, which could be heterogenic.

The phylogenetic trees from this study were comparable with species Groups (I-IV) defined by Lim et al. [30]. Group I, found in the ITS tree, includes members of the *P. pseudodelicatissima* species complex, as well as *P. fukuyoi*, *P. lundholmiae*, and *P. plurisecta*. The LSU tree recovered Group I along with *P. cuspidata*, albeit with the absence of *P. plurisecta*. The *rbc*L tree did not include the mentioned species because they were not available in GenBank. Group III was well represented in all the phylogenetic trees with *P. mannii*, *P. calliantha*, and *P. hasleana*. Group IV was represented in all the observed genetic markers and included *P. galaxiae*, *P. decipiens*, *P. simulans*, *P. allochrona*, *P. micropora*, and *P. delicatissima*. In the ITS and *rbc*L, phylogenies intraspecific differentiation was observed between *P. galaxiae* strains isolated at different study locations; this result was possibly related to environmental factors. In the North Adriatic, Turk Dermastia et al. [23] reported genetic variations between *P. galaxiae* species isolated from the same net tow.

The basal clade of the ITS tree was the *P. seriata* group, and it included *P. pungens*, *P. brasiliana*, *P. nanaoensis*, *P. americana*, *P. multiseries*, *P. australis*, *P. seriata*, and *P. multistriata*. All of the mentioned species lack central nodules, and they clustered together in accordance with Lim et al.’s findings [30]. The species *P. simulans* was found in the *P. delicatissima* complex in the ITS and LSU phylogenies, even though it has one row of poroids (synamorphy), while the *P. delicatissima* complex species have two rows of poroids. This finding corresponds with Lim et al.’s research results [30]. Members of the *Pseudo-nitzchia pseudodelicatissima* complex were best recognised in the ITS phylogenetic tree, and this result is in accordance with Lim et al.’s findings [30]. The mentioned *P. pseudodelicatissima* complex included the following species: *P. cuspidata*, *P. fukuyoi*, *P. pseudodelicatissima*, *P. plurisecta*, and *P. lundholmiae*.

The species *P. fraudulenta* and *P. subfraudulenta* were recovered in the LSU phylogenies as part of the *P. fraudulenta* group with strong nodal support. However, genetic variation was present among the isolated *P. fraudulenta* strains only in the *rbc*L phylogenies, although the *P. fraudulenta* strains were obtained from the same study location.

All the phylogenetic trees are consistent with the available phylogenetic data based on Adriatic strains for *Pseudo-nitzschia* species [23,24] as well as with the most recent studies for non-Adriatic species [6,7].

## 4. Materials and Methods

### 4.1. Study Area and Sampling

**Figure 14:**
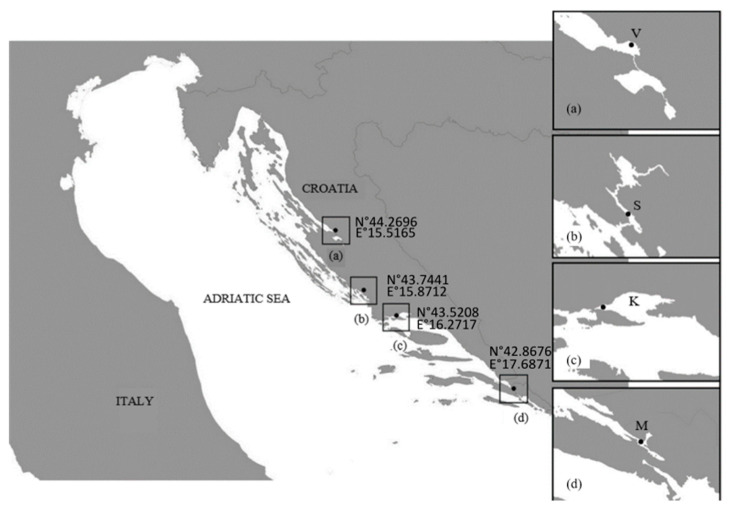
The study stations: (**a**) V—Velebit Channel, (**b**) S—Šibenik Bay, (**c**) K—Kaštela Bay, (**d**) and M—Mali Ston Bay.

The study area included the coastal regions of the Central and Southeastern Adriatic Sea. The sampling stations were as follows: (a) Velebit Channel (V; 44.2696° N, 15.5165° E), (b) Šibenik Bay (S; 43.7441° N, 15.8712° E), (c) Kaštela Bay (K; 43.5208° N, 16.2717° E), and (d) Mali Ston Bay (M; 42.8676° N, 17.6871° E) (Figure 14). Preliminary sampling was conducted in 2020 and 2021, while monthly sampling began in February 2022 and continued until February 2024. Seasons were defined as follows: winter (January–April); spring (May–June); summer (July–October); and autumn (November–December) [32]. Samples were collected using a phytoplankton net with a mesh size of 20 μm. The plankton net was towed vertically at all study stations from 5 m to the surface, except in Šibenik Bay, where the net was towed from 7 m to the surface. Temperature and salinity were measured simultaneously using a YSI Pro 1030 probe at the surface and at depths of 5 m and 7 m, as reported in Supplementary Table S2. In cases of low surface salinity due to high input/discharge of the River Krka, cell isolation was performed using samples taken at a depth of 7 m via a Niskin water sampler.

### 4.2. Cell Isolation and Cultivation

Cell cultures were established by isolating a single cell or cell chain with a drawn glass micropipette from live plankton net samples as soon as possible after sampling for the following molecular and morphological analyses. Isolation was performed using Olympus IX51 (Olympus Corporation, Tokyo, Japan) and Leica DMI 4000B (Leica Microsystems CMS, Wetzlar, Germany) inverted light microscopes at magnifications of 100× or 200×. Afterward, isolated cells were transferred sequentially to a few drops of sterile culture medium and then a 48-well plate containing up to 1 mL of culture medium. The cell cultures were maintained within a temperature range of 14 °C to 20 °C ± 0.5 °C depending on environmental conditions. Isolated cells were exposed to a 12:12 light cycle at a light intensity of 108 μmol photons m^−2^ s^−1^. The culture medium consisted of sterile filtered seawater enriched with f/2 nutrients in addition to silicates [33]. The plates containing the isolated cells were examined for growth. Subsequently, the actively growing cells were transferred to flasks containing 35 mL of enriched f/2 medium. These flasks were then kept under the same conditions as previously described to ensure their continued growth and development. Samples for molecular and morphological analyses were obtained after sufficient density had been achieved, with a maximum generally being reached after 2 to 3 weeks. The collected strains were then processed for molecular and morphological analyses. For molecular analyses, culture volumes of 6–10 mL were collected and centrifuged at 4000× *g* rpm for 20 min. The supernatant was then removed, and the pellet was transferred to a 1.5 mL sterile microcentrifuge tube and centrifuged at 13,000× *g* rpm for 10 min. The samples were then frozen until analysis. Culture samples for morphological analyses were fixed with acidic Lugol’s iodine solution at an approximate concentration of 1.5% and stored in the dark at 4 °C until further analysis. To prepare acidic Lugol’s iodine solution, 100 g of potassium iodide (KI) was dissolved in 1 L of distilled water. Following this, 50 g of solid iodine (I_2_) was added and mixed until it fully dissolved in the mixture. Afterward, 100 g of glacial acetic acid (CH_3_COOH) was mixed thoroughly. If any iodine crystals remained undissolved, the solution was decanted.

### 4.3. Morphological Analyses

Isolated *Pseudo-nitzschia* cell cultures from the field samples were analysed using a field emission scanning electron microscope equipped with a retractable STEM detector (FE-SEM/STEM Mira3, Tescan, Brno, Czech Republic). Samples were prepared for SEM analyses according to the protocol reported by Hasle and Fryxell [34]. The samples were well mixed and transferred into a beaker. An equal amount of concentrated sulfuric acid (H_2_SO_4_) was added to a mixed *Pseudo-nitzschia* culture sample. The solution was gently mixed under a hood. The saturated solution was slowly added while mixing it until the solution turned purple, indicating the oxidation of organic matter. The saturated potassium permanganate solution was prepared by adding 7–8 g of potassium permanganate (KMnO_4_) into 100 mL of distilled water. Afterwards, saturated oxalic acid ((COOH)_2_) was added, mixing the solution after each addition, until the solution slowly became transparent. The concentration of saturated oxalic acid ((COOH)_2_) was prepared by adding 10 g of oxalic acid ((COOH)_2_) into 100 mL of distilled water. The sample was rinsed with deionised water through repeated centrifugation at 3000× *g* rpm for 20 min until the pH was close to neutral. Cleaned samples were filtered through polycarbonate filters with a pore size of 1 μm and a diameter of 13 mm and dried in a desiccator for at least 24 h (Nucleopore, Whatman, Maidstone, UK). Filters were then mounted on aluminium stubs, which were gold-coated using a sputter coater (Quorum Technologies, Q150RES, Lewes, UK) for scanning electron microscopy. SEM analyses were performed at an accelerating voltage of 4 kV and a working distance of 4 mm. Additionally, some cultures were analysed with a STEM detector at an accelerating voltage in the range of 7–9 kV and a working distance ranging from 3–6 mm, depending on the analysed species. For STEM analysis, 10–20 μL of cleaned culture sample was gently put on TEM mesh (FCF200-NI, 200 mesh, formvar/carbon coated nickel grid, Electron Microscopy Sciences, Hatfield, PA, USA). For morphological analyses, at least 10 cells were measured for each culture, accounting for width, length, number of fibulae and interstriae in 10 μm, number of poroids in 1 μm, rows of poroids, poroid sectors, and band striae in 10 μm.

### 4.4. DNA Extraction, PCR Amplification, and Sequencing

Total genomic DNA was extracted from cultures (app. volume 6–10 mL) in the exponential growth phase using the DNeasy Plant Mini Kit (Qiagen, Gilden, Germany) according to the manufacturer’s guidelines. The concentration of extracted DNA was measured with a NanoDrop spectrophotometer (Thermo Fisher). PCR amplification was performed using either recombinant Q5 Hot-Start High-Fidelity Master Mix (New England Biolabs, Ipswich, MA, USA) or Phire Tissue Direct PCR Master Mix (Thermo Scientific) in a total volume of 25 μL according to the manufacturer’s instructions. PCR reaction contained Master Mix, 0.02 μM of each primer, 1 ng/µL of genomic DNA template, and UltraPure DNase/RNase-Free Distilled Water (Invitrogen, Thermo Fisher Scientific, Waltham, MA, USA). DNA was amplified using a series of primers (details of PCR protocols are shown in Supplementary Table S3). To confirm the *Pseudo-nitzschia* species, three different regions were amplified: the entire internal transcribed spacer ITS (ITS1/5.8S/ITS2), the LSU region of the DNA (large subunit), and *rbc*L (ribulose-1,5-biphosphate carboxylase/oxidase). The PCR products were visualised under UV light using 1% agarose gel electrophoresis. Purification and Sanger sequencing of PCR products were performed by Macrogene Europe (Amsterdam, The Netherlands). For the ITS amplification, the forward and reverse primers combined were PSNF1 (5′GGATCATTACCACACCGATCC3′) and PSNR1 (5′CCTCTTGCTTGATCTGAGATCC3′) [35] and ITSA (5′CCAAGCTTCTAGATCGTAACAAGGTCCGTAGGT3′) combined with ITSB primer (5′CCTGCAGTCGACAATGCTTAATTCAGCGG3′) [36]. For LSU rDNA amplification, the primers used were D1R (5′ACCCGCTGAATTTAAGCATA3′) and D3B (5′TCGGAGGGAACCAGCTACTA3′) [37,38] as well as D1R (5′ACCCGCTGAATTTAAGCATA3′) [37] and D3Ca (ACGAACGATTTGCACGTCAG3′). The *rbc*L gene region was amplified using the primers *rbc*L1 (5′AAGGAGAAATHAATGTCT3′) and *rbc*L7 (5′AARCAACCTTGTGTAAGTCT3′) [39].

### 4.5. Sequence Analysis and Phylogeny

Chromatograms were assembled and quality-checked using Chromas Pro (v2.1.10.1). For molecular species identification, the ITS, LSU rDNA, and *rbc*L sequences were compared with *Pseudo-nitzschia* spp. sequences published in the National Center for Biotechnology Information (NCBI) nucleotide database using BLASTn (NCBI) [40]. A sequence is confirmed as corresponding to a species if the similarity percentage is over 99%. Gene markers (ITS, LSU, and *rbc*L) were analysed separately to obtain distinct phylogenies. Alignments were made using CLUSTALW (version 2.1) with MEGA11 (version 11.0.13) software using default settings [41,42]. The resulting ITS, LSU, and *rbc*L alignments had 738, 712, and 1365 nucleotides, respectively. We developed the evolutionary model of nucleotide substitution and calculated the shape parameters of the gamma distribution using Bayesian information criteria integrated in MEGA11 [41,42]. Bayesian Inference (BI) was made with a generalised time-reversible evolution model (GTR) with gamma distribution and invariant sites (G+I) for BI for all barcodes [43]. Pre-aligned FASTA file was used as input for Bayesian analyses, which were carried out using MrBayes 3.2.7 [44] with a minimum of 4,000,000 Markov chain Monte Carlo generations with a sample frequency of 1000 generations and diagnostic frequencies of 1000 until the average standard deviation fell between 0.01 and 0.05. Additionally, a combined phylogenetic tree was created using the ITS, LSU, and *rbc*L alignments, which were merged using R-Studio software (version 4.3.0) [45], version 4.3.0 with packages magrittr and dplyr from CRAN Repository (RStudio, PBC). The construction of BI for the combined phylogenetic tree was conducted with a GTR+G+I model with a minimum of 10,000,000 Markov chain Monte Carlo generations with a sample frequency of 1000 generations and diagnostic frequencies of 1000 until the standard deviation fell between 0.01 and 0.05. Outputs for ITS, LSU, *rbc*L, and combined phylogenetic tree were visualised using FigTree. The first 25% of samples from the cold chain were discarded by default. All posterior probabilities above 0.85 are shown. The genetic pair-wise values and overall pair-wise values were calculated in MEGA11 [41,42]. All downloaded sequences used in creating phylogenetic trees have GenBank accession numbers next to the species names. *Nitzschia navis-varingica* (GenBank accession number: KX353643.1) was used as the outgroup for conducting ITS phylogenies, while for LSU and *rbc*L, the outgroup used was *Cylindrotheca* sp. (GenBank accession numbers OM9360007.1 and M59080.1, respectively). The outgroup used in the analysis for the combined phylogenetic tree was *Cylindrotheca closterium* (GenBank accession number: NC_037986.1). The selected imported sequences for the combined phylogenetic tree were gathered from studies conducted in the same location and time period.

## 5. Conclusions

In conclusion, an approach combining molecular and morphological methods provides reliable and accurate insights into the composition of *Pseudo-nitzschia* species. Our results are especially significant as the reported areas are important aquaculture areas, and the findings will contribute substantially to the accurate identification of *Pseudo-nitzschia* species in these regions. However, we are aware that the analysis of cell cultures cannot give a complete insight into the native community of *Pseudo-nitzschia* diversity because of different shortcomings: the sampling method itself, the isolation of the cells, and the sensitivity of *Pseudo-nitzschia* species under the culture conditions. Nevertheless, the genetic characterisation analysis of cell cultures is crucial and very informative for the differentiation of cryptic and pseudo-cryptic species within the genus *Pseudo-nitzschia*. The findings of our study will make an important contribution to the development of the database for the discussed genetic sequences, especially for the *rbc*L marker. The establishment of sequence databases is necessary for the use of new methods, such as metabarcoding, and will serve our future phytoplankton biodiversity research.

## Figures and Tables

**Table 1:** List of species identified with the corresponding information about the isolation location, season (SP: spring, SU: summer, A: autumn, and W: winter), and species confirmation method.

Species	Number of Cultures	Location	Season	Methods
*P. allochrona*	14	K, M, S	A, SU	ITS, LSU, *rbc*L, SEM
*P. calliantha*	24	All locations	A, W, SU	ITS, LSU, *rbc*L, SEM/STEM
*P. delicatissima*	47	K, M, V	A, W, SP	ITS, LSU, *rbc*L, SEM/STEM
*P. fraudulenta*	6	K	A, W	ITS, LSU, *rbc*L, SEM
*P. mannii*	51	All locations	All seasons	ITS, LSU, *rbc*L, SEM/STEM
*P. multistriata*	7	S	A	ITS, LSU, *rbc*L, SEM/STEM
*P. pseudodelicatissima*	16	K	A, W, SU	ITS, LSU, *rbc*L, SEM/STEM
*P. subfraudulenta*	6	S, V	A, SU	ITS, LSU, *rbc*L, SEM/STEM

**Table 2:** Morphometric measurements made for *Pseudo-nitzschia* species. Data are given as minimum and maximum ranges in bold, with average values ± standard deviation specified below. The number of measurements (*n*) and analysed strains are specified in parentheses.

Species	Width (µm)	Length (µm)	Central Interspace	Fibulae in 10 µm	Interstriae in 10 µm	Poroids in 1 µm	Rows of Poroids	Poroid Sectors	Band Striae in 10 µm
*P. allochrona* (*n* = 50; 5)	**1.44–2.15** 1.72 ± 0.18	**51.06–63.01** 58.12 ± 3.58	+	**20–24** 21.6 ± 0.9	**36–39** 37.1 ± 0.8	**7–13**	(1)-2	-	**43–45** 43.9 ± 0.7
*P. calliantha* (*n* = 97; 9)	**1.38–2.12** 1.75 ± 0.17	**64.08–107.14** 83.53 ± 12.11	+	**17–22** 19.6 ± 1.1	**34–38** 36.2 ± 0.8	**4–6**	1	2–11 * 6	**43–46** 44.5 ± 0.8
*P. delicatissima* (*n* = 126; 12)	**1.31–2.00** 1.64 ± 0.15	**31.05–94.21** 57.43 ± 17.00	+	**20–26** 23.3 ± 1.2	**37–42** 40.4 ± 0.7	**7–13**	(1)-2	-	**48–51** 49.6 ± 0.6
*P. fraudulenta* (*n* = 56; 5)	**5.09–6.54** 5.79 ± 0.37	**60.22–67.42** 65.04 ± 1.45	+	**19–24** 22.7 ± 0.9	**23–25** 23.8 ± 0.6	**5–7**	(1)-2-(3)	2–7 * 5	**38–41** 39.5 ± 0.6
*P. mannii* (*n* = 148; 14)	**1.71–2.48** 2.05 ± 0.16	**69.08–123.56** 93.37 ± 13.37	+	**16–23** 19.7 ± 1.2	**33–37** 35.3 ± 0.7	**4–6**	1	1–8 * 4	**42–44** 43.2 ± 0.6
*P. multistriata* (*n* = 62; 6)	**2.33–3.51** 2.91 ± 0.30	**55.85–84.85** 68.07 ± 7.94	-	**23–26** 24.3 ± 0.8	**36–40** 38.4 ± 0.9	**9–12**	(1)-2	-	**47–51** 49.9 ± 0.9
*P. pseudodelicatissima* (*n* = 84; 8)	**1.22–1.75** 1.48 ± 0.12	**59.14–90.99** 72.76 ± 11.06	+	**19–26** 22.3 ± 1.2	**38–41** 39.3 ± 0.7	**5–7**	1	1–4 * 2	**47–52** 49.6 ± 1.0
*P. subfraudulenta* (*n* = 60; 5)	**4.15–5.25** 4.69 ± 0.22	**96.19–140.33** 118.42 ± 15.98	+	**13–17** 14.8 ± 0.9	**23–25** 24.6 ± 0.5	**5–6**	(1)-2	2–10 * 5	**39–41** 40.1 ± 0.6

* The number of measurements for poroid sectors are as follows: *P. calliantha n* = 2638, *P. fraudulenta n* = 507, *P. mannii n* = 4103, *P. pseudodelicatissima n* = 1347, and *P. subfraudulenta n* = 1416.

## Data Availability

Dataset can be made available by the authors upon request.

## Supplementary Materials

The following supporting information can be downloaded at https://www.mdpi.com/article/10.3390/plants14020245/s1. Table S1: List of *Pseudo-nitzschia* species based on location (V—Velebit Channel, S—Šibenik Bay, K—Kaštela Bay, and M—Mali Ston Bay), year of isolation, and accession numbers of ITS, LSU, and *rbc*L sequences deposited in GenBank. Table S2: Seasonal temperature and salinity range (min–max) measured during the study period at the studied areas: V—Velebit Channel, S—Šibenik Bay, K—Kaštela Bay, and M—Mali Ston Bay. Table S3: Set of primers used for amplification of ITS, LSU, and *rbc*L barcode according to specific PCR protocols.
