# Supplementary material for: Advancing the Taxonomy of the Diatom Pseudo-nitzschia Through an Integrative Study Conducted in the Central and Southeastern Adriatic Sea
Source: Plants (Basel). 2025 Jan 16;14(2):245. doi: 10.3390/plants14020245 (PMC11768262; doi:10.3390/plants14020245)
Supplement: Supplementary file 1 [file plants-14-00245-s001.zip › plants-3409649-supplementary.pdf]

Supplementary data

**Supplementary Table S1.** List of *Pseudo-nitzschia* species based on location (V- Velebit Channel, S- Šibenik Bay, K- Kaštela Bay, M- Mali Ston Bay, year of isolation, ITS, LSU, and rbcL accession number (GenBank).

| ID strain | Species | Location | Year of isolation | LSU accession number (GenBank) | ITS accession number (GenBank) | rbcL accession number (GenBank) |
|---|---|---|---|---|---|---|
| K055al | *P. allochrona* | K | 2021 | OQ319829 | OQ309060 | / |
| K273al | *P. allochrona* | K | 2022 | PP215940 | PP10944 | PQ351355 |
| K341al | *P. allochrona* | K | 2022 | PP215950 | PP109426 | PP213344 |
| M326al | *P. allochrona* | M | 2022 | PP215995 | PP109424 | PP213355 |
| S018al | *P. allochrona* | S | 2021 | OQ319822 | OQ309053 | PP213360 |
| S019al | *P. allochrona* | S | 2021 | OQ319823 | OQ309054 | PP213361 |
| S020al | *P. allochrona* | S | 2021 | OQ319824 | OQ309055 | / |
| S021al | *P. allochrona* | S | 2021 | OQ319825 | OQ309056 | PP213362 |
| S022al | *P. allochrona* | S | 2021 | OQ319826 | OQ309057 | PP213363 |
| S024al | *P. allochrona* | S | 2021 | OQ319827 | OQ309058 | PP213365 |
| S029al | *P. allochrona* | S | 2021 | OQ319828 | OQ309059 | PP213364 |
| S222al | *P. allochrona* | S | 2022 | PP216012 | PP109422 | PP213366 |
| S223al | *P. allochrona* | S | 2022 | PP216013 | PP109423 | PP213367 |
| S280al | *P. allochrona* | S | 2022 | PP216014 | PP109425 | PP213368 |
| K111ca | *P. calliantha* | K | 2022 | PP215933 | PP109427 | PP213333 |
| K131ca | *P. calliantha* | K | 2022 | OQ309019 | OQ309036 | PP213335 |
| K132ca | *P. calliantha* | K | 2022 | OQ309022 | OQ309037 | PP213336 |
| K135ca | *P. calliantha* | K | 2022 | OQ309023 | OQ309029 | / |
| K274ca | *P. calliantha* | K | 2022 | PP215941 | PP126544 | PP213339 |
| K367ca | *P. calliantha* | K | 2023 | PP215960 | PP109430 | PP213347 |
| M074ca | *P. calliantha* | M | 2022 | OQ309026 | OQ309033 | PP213391 |
| M091ca | *P. calliantha* | M | 2022 | OQ309025 | OQ309035 | / |
| S354ca | *P. calliantha* | S | 2022 | PP216025 | PP109450 | PQ351385 |
| V008ca | *P. calliantha* | V | 2020 | OQ309020 | OQ309028 | PP213375 |
| V013ca | *P. calliantha* | V | 2020 | OQ309021 | OQ309030 | PP213376 |
| V014ca | *P. calliantha* | V | 2020 | OQ309018 | OQ309031 | PP213377 |
| V062ca | *P. calliantha* | V | 2022 | OQ309027 | OQ309032 | / |
| V068ca | *P. calliantha* | V | 2022 | PQ063246 | PP109445 | / |
| V070ca | *P. calliantha* | V | 2022 | PQ002445 | PP109446 | PP213385 |
| V071ca | *P. calliantha* | V | 2022 | PQ063247 | PP109447 | / |
| V072ca | *P. calliantha* | V | 2022 | PQ063248 | PP109448 | / |
| V077ca | *P. calliantha* | V | 2022 | PQ002442 | PP109429 | PP213386 |
| V227ca | *P. calliantha* | V | 2022 | PQ063249 | PP109444 | / |
| V283ca | *P. calliantha* | V | 2022 | PQ002443 | PP126545 | PP213389 |
| V285ca | *P. calliantha* | V | 2022 | PP215942 | PP109449 | PP213369 |
| V299ca | *P. calliantha* | V | 2022 | PQ002446 | PP109428 | PP213354 |
| V437ca | *P. calliantha* | V | 2023 | PQ002444 | PQ002610 | PQ351387 |
| V079ca | *P. calliantha* | V | 2022 | OQ309024 | OQ309034 | PP213387 |
| K057de | *P. delicatissima* | K | 2021 | OQ309088 | OQ309040 | / |
| K058de | *P. delicatissima* | K | 2021 | OQ309098 | OQ309042 | PP213329 |
| K129de | *P. delicatissima* | K | 2022 | OQ309091 | OQ309044 | PP213330 |
| K134de | *P. delicatissima* | K | 2022 | OQ309092 | OQ309045 | PP213337 |
| K159de | *P. delicatissima* | K | 2022 | OQ309096 | OQ309049 | PQ366017 |

| | | | | | | |
|---|---|---|---|---|---|---|
| K368de | *P. delicatissima* | K | 2023 | PP215961 | PP126525 | / |
| K369de | *P. delicatissima* | K | 2023 | PP215962 | PP126526 | / |
| K370de | *P. delicatissima* | K | 2023 | PP215963 | PP126527 | / |
| K371de | *P. delicatissima* | K | 2023 | PP215964 | PP126528 | / |
| K372de | *P. delicatissima* | K | 2023 | PP215965 | PP126529 | / |
| K373de | *P. delicatissima* | K | 2023 | PP215966 | PP126530 | / |
| K374de | *P. delicatissima* | K | 2023 | PP215967 | PP126531 | / |
| K376de | *P. delicatissima* | K | 2023 | PP215968 | PP126543 | PQ351366 |
| K377de | *P. delicatissima* | K | 2023 | PP215969 | PP126533 | / |
| K378de | *P. delicatissima* | K | 2023 | PP215970 | PP126534 | / |
| K379de | *P. delicatissima* | K | 2023 | PP215971 | PP126535 | / |
| K381de | *P. delicatissima* | K | 2023 | PP215972 | PP126536 | / |
| K399de | *P. delicatissima* | K | 2023 | PP215975 | PP126537 | / |
| K408de | *P. delicatissima* | K | 2023 | PP215976 | PP126538 | / |
| K421de | *P. delicatissima* | K | 2023 | PP215978 | PP126540 | / |
| K423de | *P. delicatissima* | K | 2023 | PP215979 | PP126541 | / |
| K424de | *P. delicatissima* | K | 2023 | PP215980 | PP126542 | / |
| K452de | *P. delicatissima* | K | 2023 | PQ002447 | PQ002611 | PQ351369 |
| K454de | *P. delicatissima* | K | 2023 | PQ002448 | PQ002612 | PQ351370 |
| K459de | *P. delicatissima* | K | 2023 | PQ002449 | PQ002613 | PQ351374 |
| K465de | *P. delicatissima* | K | 2024 | PQ002470 | PQ002627 | PQ351375 |
| K481de | *P. delicatissima* | K | 2024 | PQ002462 | PQ002624 | PQ351376 |
| K484de | *P. delicatissima* | K | 2024 | PQ063250 | PQ373929 | / |
| K485de | *P. delicatissima* | K | 2024 | PQ002463 | PQ002625 | PQ351377 |
| K486de | *P. delicatissima* | K | 2024 | PQ002464 | PQ373930 | PQ351389 |
| M075de | *P. delicatissima* | M | 2022 | OQ309095 | OQ309048 | PP213349 |
| M085de | *P. delicatissima* | M | 2022 | OQ309090 | OQ309043 | PP213351 |
| M088de | *P. delicatissima* | M | 2022 | OQ309097 | OQ309050 | PQ366018 |
| M138de | *P. delicatissima* | M | 2022 | OQ309093 | OQ309046 | PQ366019 |
| M140de | *P. delicatissima* | M | 2022 | OQ309094 | OQ309047 | / |
| M144de | *P. delicatissima* | M | 2022 | OQ309089 | OQ309041 | PQ366020 |
| M363de | *P. delicatissima* | M | 2022 | PP215996 | PP126524 | / |
| M364de | *P. delicatissima* | M | 2022 | PP215997 | PP109431 | PP213356 |
| M375de | *P. delicatissima* | M | 2023 | PP215998 | PP126532 | PQ351378 |
| M415de | *P. delicatissima* | M | 2023 | PP216003 | PP126539 | PQ351379 |
| M461de | *P. delicatissima* | M | 2024 | PQ002450 | PQ002614 | PQ351380 |
| M462de | *P. delicatissima* | M | 2024 | PQ063251 | PQ373931 | / |
| V040de | *P. delicatissima* | V | 2021 | OQ309087 | OQ309038 | PP213381 |
| V041de | *P. delicatissima* | V | 2021 | OQ309086 | OQ309039 | PP213382 |
| V042de | *P. delicatissima* | V | 2021 | OQ309099 | OQ309051 | PP213383 |
| V043de | *P. delicatissima* | V | 2021 | OQ309085 | OQ309052 | PP213384 |
| V443de | *P. delicatissima* | V | 2023 | PQ063252 | / | PQ366021 |
| K450fr | *P. fraudulenta* | K | 2023 | PQ002451 | PQ002615 | PQ351367 |
| K451fr | *P. fraudulenta* | K | 2023 | PQ002455 | PQ002619 | PQ351368 |
| K455fr | *P. fraudulenta* | K | 2023 | PQ002452 | PQ002616 | PQ351371 |
| K456fr | *P. fraudulenta* | K | 2023 | PQ002453 | PQ002617 | PQ351372 |
| K458fr | *P. fraudulenta* | K | 2023 | PQ002454 | PQ002618 | PQ351373 |
| K468fr | *P. fraudulenta* | K | 2024 | PQ063253 | PQ373932 | / |
| K136ga | *P. galaxiae* | K | 2022 | OQ312051 | OQ309100 | PP213338 |
| M232ga | *P. galaxiae* | M | 2022 | OQ983983 | OQ983978 | PP213353 |
| K056ha | *P. hasleana* | K | 2021 | OQ309152 | OQ309102 | PP213328 |
| K082ha | *P. hasleana* | K | 2022 | OQ309153 | OQ309099 | PP213331 |
| K083ha | *P. hasleana* | K | 2022 | OQ309154 | OQ309103 | PP213327 |

| | | | | | | |
|---|---|---|---|---|---|---|
| K101ha | *P. hasleana* | K | 2022 | OQ309155 | OQ309104 | PP213332 |
| K115ha | *P. hasleana* | K | 2022 | OQ309156 | OQ309105 | PP213334 |
| K026ma | *P. mannii* | K | 2021 | OQ319199 | OQ309133 | / |
| K103ma | *P. mannii* | K | 2022 | OQ319208 | OQ309142 | PQ366022 |
| K128ma | *P. mannii* | K | 2022 | OQ319209 | OQ309143 | / |
| K130ma | *P. mannii* | K | 2022 | OQ319210 | OQ309144 | / |
| K216ma | *P. mannii* | K | 2022 | PP215936 | PQ373933 | / |
| K231ma | *P. mannii* | K | 2022 | PQ002457 | PP109453 | PQ366023 |
| K237ma | *P. mannii* | K | 2022 | OQ319216 | OQ309150 | PQ366024 |
| K260ma | *P. mannii* | K | 2022 | PP215939 | PP109458 | / |
| K321ma | *P. mannii* | K | 2022 | PP215944 | PP109433 | PP213340 |
| K334ma | *P. mannii* | K | 2022 | PP215947 | PP109460 | PP213342 |
| K397ma | *P. mannii* | K | 2023 | PP215974 | PP109461 | / |
| K410ma | *P. mannii* | K | 2023 | PP215977 | PP109463 | / |
| K478ma | *P. mannii* | K | 2024 | PQ063254 | / | PQ366025 |
| M002ma | *P. mannii* | M | 2020 | PP215981 | PP109466 | / |
| M003ma | *P. mannii* | M | 2020 | OQ319196 | OQ309130 | PP213348 |
| M033ma | *P. mannii* | M | 2021 | OQ319200 | OQ309137 | / |
| M037ma | *P. mannii* | M | 2021 | OQ319201 | OQ309134 | / |
| M039ma | *P. mannii* | M | 2021 | OQ319202 | OQ309135 | / |
| M073ma | *P. mannii* | M | 2022 | OQ319204 | OQ309138 | / |
| M084ma | *P. mannii* | M | 2022 | PP215986 | PP109436 | PP213350 |
| M094ma | *P. mannii* | M | 2022 | PQ002466 | PP109434 | PP213352 |
| M141ma | *P. mannii* | M | 2022 | OQ319214 | OQ309145 | / |
| M150ma | *P. mannii* | M | 2022 | OQ319211 | OQ309146 | / |
| M153ma | *P. mannii* | M | 2022 | / | PQ373934 | PQ366026 |
| M205ma | *P. mannii* | M | 2022 | PP215988 | PP109451 | / |
| M208ma | *P. mannii* | M | 2022 | PP215989 | PP109452 | / |
| M219ma | *P. mannii* | M | 2022 | PQ002467 | / | PQ366027 |
| M233ma | *P. mannii* | M | 2022 | PP215991 | PP109454 | PQ366028 |
| M239ma | *P. mannii* | M | 2022 | OQ319217 | OQ309151 | / |
| M240ma | *P. mannii* | M | 2022 | PP215993 | PP109456 | PQ366029 |
| M241ma | *P. mannii* | M | 2022 | PP215994 | PP109457 | / |
| M380ma | *P. mannii* | M | 2023 | PP215999 | PP109459 | / |
| M400ma | *P. mannii* | M | 2023 | PP216000 | PP109465 | / |
| M401ma | *P. mannii* | M | 2023 | PP216001 | PP109480 | / |
| M404ma | *P. mannii* | M | 2023 | PP216002 | PP109462 | / |
| M435ma | *P. mannii* | M | 2023 | PP216004 | PP109481 | / |
| M463ma | *P. mannii* | M | 2024 | PQ002461 | PQ002623 | PQ351381 |
| S155ma | *P. mannii* | S | 2022 | OQ319212 | OQ309147 | PQ366030 |
| S156ma | *P. mannii* | S | 2022 | PQ002468 | PP109435 | PP213357 |
| S157ma | *P. mannii* | S | 2022 | OQ319213 | OQ309148 | PQ351382 |
| S383ma | *P. mannii* | S | 2023 | PP215927 | PP109467 | / |
| S385ma | *P. mannii* | S | 2023 | PP215928 | PP109464 | PP213374 |
| S393ma | *P. mannii* | S | 2023 | PP215973 | PP109468 | / |
| V015ma | *P. mannii* | V | 2020 | PQ002456 | PP126522 | PP213378 |
| V016ma | *P. mannii* | V | 2020 | OQ319198 | OQ309132 | PP213379 |
| V017ma | *P. mannii* | V | 2020 | PQ002469 | PP109432 | PP213380 |
| V063ma | *P. mannii* | V | 2022 | OQ319203 | OQ309136 | / |
| V078ma | *P. mannii* | V | 2022 | OQ319205 | OQ309139 | PQ366031 |
| V296ma | *P. mannii* | V | 2022 | PP216020 | PP109485 | / |
| V439ma | *P. mannii* | V | 2022 | PQ002460 | PQ002622 | PQ351388 |
| S004mu | *P. multistriata* | S | 2020 | OQ319167 | OQ309016 | PP213358 |

| | | | | | | |
|---|---|---|---|---|---|---|
| S005mu | *P. multistriata* | S | 2020 | OQ319168 | OQ309017 | PP213359 |
| S290mu | *P. multistriata* | S | 2022 | PP216017 | PP109438 | PP213370 |
| S294mu | *P. multistriata* | S | 2022 | PP216019 | PP109469 | / |
| S300mu | *P. multistriata* | S | 2022 | PP216022 | PP126547 | PP213372 |
| S302mu | *P. multistriata* | S | 2023 | PP216023 | PP109437 | PP213373 |
| S442mu | *P. multistriata* | S | 2023 | PQ002458 | PQ002620 | PQ351386 |
| K028ps | *P. pseudodelicatissima* | K | 2021 | OQ318554 | OQ318552 | / |
| K319ps | *P. pseudodelicatissima* | K | 2022 | PP215943 | PQ002621 | PQ351356 |
| K328ps | *P. pseudodelicatissima* | K | 2022 | PP216024 | PP109439 | PQ351357 |
| K333ps | *P. pseudodelicatissima* | K | 2022 | PQ002465 | PQ002626 | PQ351358 |
| K336ps | *P. pseudodelicatissima* | K | 2022 | PP215948 | PP109440 | PP213343 |
| K339ps | *P. pseudodelicatissima* | K | 2022 | PQ063255 | / | PQ366034 |
| K340ps | *P. pseudodelicatissima* | K | 2022 | PP215949 | PP109470 | / |
| K349ps | *P. pseudodelicatissima* | K | 2022 | PP215951 | PP109441 | PP213345 |
| K350ps | *P. pseudodelicatissima* | K | 2022 | PP215952 | PP109442 | PP213346 |
| K351ps | *P. pseudodelicatissima* | K | 2022 | PP215953 | PP109477 | PQ351359 |
| K352ps | *P. pseudodelicatissima* | K | 2022 | PP215954 | PP109471 | PQ351360 |
| K356ps | *P. pseudodelicatissima* | K | 2022 | PP215955 | PP109472 | PQ351361 |
| K357ps | *P. pseudodelicatissima* | K | 2022 | PP215956 | PP109473 | PQ351362 |
| K358ps | *P. pseudodelicatissima* | K | 2022 | PP215957 | PP109474 | PQ351363 |
| K359ps | *P. pseudodelicatissima* | K | 2022 | PP215958 | PP109475 | PQ351364 |
| K366ps | *P. pseudodelicatissima* | K | 2023 | PP215959 | PP109476 | PQ351365 |
| S287su | *P. subfraudulenta* | S | 2022 | PP216015 | PP109482 | PQ351383 |
| S288su | *P. subfraudulenta* | S | 2022 | PP216016 | PP109483 | PQ351384 |
| S291su | *P. subfraudulenta* | S | 2022 | PP216018 | PP109484 | / |
| S297su | *P. subfraudulenta* | S | 2022 | PP216021 | PP126546 | PP213371 |
| V253su | *P. subfraudulenta* | V | 2022 | PQ063257 | / | PQ366033 |
| V284su | *P. subfraudulenta* | V | 2022 | PQ002459 | PP126523 | PP213390 |

**Supplementary Table S2.** Seasonal temperature and salinity range (min-max) measured during the study period at the studied areas: V-Velebit Channel, S-Šibenik Bay, K-Kaštela Bay, M- Mali Ston Bay.

| | | Temperature (°C) | | Salinity | |
|---|---|---|---|---|---|
| | | 0 m | 5 m | 0 m | 5 m |
| Winter | V | 7.3-14.2 | 10.0-16.0 | 20.1-37.0 | 35.2-37.8 |
| | S | 6.3-11.9 | 13.8-17.1 | 2.7-17.3 | 36.6-38.3 |
| | K | 11.0-14.0 | 11.5-14.5 | 31.1-36.0 | 35.8-37.1 |
| | M | 9.8-16.8 | 10.4-16.9 | 34.3-38.4 | 35.2-38.5 |
| Spring | V | 14.0-21.0 | 12.6-17.9 | 18.2-32.3 | 36.1-36.6 |
| | S | 16.4-21.9 | 15.9-20.5 | 3.6-12.3 | 36.5-37.9 |
| | K | 16.1-22.1 | 16.8-21.5 | 29.4-34.0 | 35.8-36.9 |
| | M | 17.6-20.6 | 15.4-18.5 | 30.0-36.3 | 36.5-38.1 |
| Summer | V | 18.8-27.3 | 19.0-25.5 | 28.1-36.6 | 35.0-37.1 |
| | S | 19.8-27.0 | 19.4-26.4 | 11.4-28.8 | 33.6-38.9 |
| | K | 20.2-27.1 | 20.4-27.1 | 34.0-37.6 | 36.6-38.7 |
| | M | 19.4-28.1 | 20.0-27.6 | 31.0-36.6 | 33.0-37.2 |
| Autumn | V | 11.7-16.6 | 15.2-18.1 | 14.1-36.6 | 36.0-37.7 |
| | S | 10.6-18.1 | 18.8-21.8 | 4.1-26.6 | 36.8-38.8 |
| | K | 12.9-19.2 | 15.9-20.5 | 27.5-37.1 | 36.2-38.0 |
| | M | 13.9-19.1 | 16.2-19.3 | 32.0-38.3 | 35.9-38.3 |

**Supplementary Table S3.** Set of primers used for amplification of ITS, LSU, and rbcL barcode according to specific PCR protocols.

| Enzyme | Marker | Primer set | Primer reference | Initialization | Denaturation | Annealing | Elongation | Final Elongation | Hold |
|---|---|---|---|---|---|---|---|---|---|
| Q5 Hot Start | ITS | PSNF1/PSNR1 | Noyer et al. [35] | 98 °C 3 min | 98 °C 10 s | 58 °C 30 s | 72 °C 30 s [2-4 35x] | 72 °C 10 min | 4 °C ∞ |
| Q5 Hot Start | ITS | ITSA/ITSB | Adachi et al. [36] | 95 °C 10 min | 94 °C 30 s | 55 °C 30 s | 72 °C 30 s [2-4 35x] | 72 °C 10 min | 4 °C ∞ |
| Q5 Hot Start | LSU | D1R/D3B | Nunn et al. [38]; Scholin et al. [37] | 98 °C 30 s | 98 °C 10 s | 52 °C 30 s | 72 °C 30 s [2-4 35x] | 72 °C 2 min | 4 °C ∞ |
| Q5 Hot Start | LSU | D1R/D3Ca | Scholin et al. [37] | 98 °C 30 s | 98 °C 10 s | 52 °C 30 s | 72 °C 30 s [2-4 35x] | 72 °C 2 min | 4 °C ∞ |
| Q5 Hot Start | rbcL | rbcL1/rbcL7 | Jones et al. [39] | 98 °C 3 min | 98 °C 10 s | 52 °C 30 s | 72 °C 30 s [2-4 35x] | 72 °C 10 min | 4 °C ∞ |
